# The Impact of Cellular Therapies on Gastrointestinal Diseases: Applications and Challenges

**DOI:** 10.5152/tjg.2023.23137

**Published:** 2023-08-01

**Authors:** Meng-Meng Zhang, Yan Hu, Jing Xu, Ling Liu, Lu-Lu Lv

**Affiliations:** 1 Department of Gastroenterology, Hangzhou Shangcheng District People’s Hospital, Hangzhou, Zhejiang Province, China; 2 Department of Gastroenterology, The Second Affiliated Hospital, Zhejiang University Faculty of Medicine, Hangzhou, Zhejiang Province, China; 3 Department of Gastroenterology, Shengzhou People’s Hospital (the First Affiliated Hospital of Zhejiang University Shengzhou Branch), Zhejiang University, Shengzhou, Zhejiang Province, China

**Keywords:** Cellular therapy, Crohn’s disease, coeliac disease, ulcerative colitis, inflammatory bowel diseases

## Abstract

Gastrointestinal diseases are highly prevalent, and their burden significantly impacts the quality of life of affected individuals. Inflammatory and immune-mediated intestinal diseases usually have a chronic course without adequate therapeutic modalities. Although much has been reported to comprehend these diseases, many remain resistant and refractory to conventional treatment approaches. Therefore, recent approaches to cellular therapy using stem cells, like hematopoietic stem cells and mesenchymal stem cells, and other cellular immunosuppressive modalities, like T-regulatory cells, were introduced and investigated in treating gastrointestinal diseases. We aimed to conduct a literature review to discuss the applications and challenges of cellular therapeutics in gastrointestinal diseases. Evidence from published clinical trials supports the safety and efficacy of cellular treatment in different immune-mediated and inflammatory gastrointestinal diseases. They can offer a longer duration of remission, being able to adjust the dysregulated immune system. However, there are various challenges to be considered by future trials, including the limitations of current clinical trials, challenges in retrieval and application of these therapeutics, and their mutagenesis potential.

**Main Points**

- Cellular therapy remains a novel technique that needs to be further studied to comprehend various factors associated with its application in gastrointestinal diseases. For instance, limitations regarding immunogenicity and adverse events of stem cells and others regarding the purification and isolation process should be addressed.
- Future studies should aim at understanding the long-term efficacy and safety and the mechanism of cellular therapy to boost the therapeutic potential of these modalities in the different immune-mediated and inflammatory intestinal diseases.
- Anticipated findings from the ongoing phase III clinical trials might overcome these challenges and provide insight standardized application of stem cells. Using cellular therapy by other modalities, rather than hematopoietic and mesenchymal stem cells, should also be encouraged and investigated by preclinical models.

## Introduction

Therapeutic stem cells, like hematopoietic stem cells (HSCs) and mesenchymal stem cells (MSCs), have recently been introduced as effective treatments for different medical conditions.^1^ The efficacy of these modalities owes to their proven abilities to differentiate into many types and degrees of progenesis, representing the basic structures of different human body tissues. Self-renewal, multiple functions, and indefinite increase also characterize them. Various stem cell types have been validated and can be stratified into 3 main types: adult, induced multipotential, and embryonic stem cells (ESCs).^2^

Applying stem cell therapy for treating gastrointestinal conditions has gained attention within the past few years because of the remarkable outcomes reported for these modalities in clinical trials.^3,4^ Stem cells are mainly applied to treat immune-mediated and inflammatory gastrointestinal disorders, like inflammatory bowel disease (IBD), including Crohn’s disease and ulcerative colitis.^5,6^ Moreover, various advantages have been proposed for these modalities compared to other therapeutic approaches (Table 1). Therefore, stem cell therapy has recently advanced in this field and gained much attention from the industry. Currently, HSCs and MSCs are the mainly applied cellular therapeutics to treat gastrointestinal diseases. In this literature review, we will discuss the applications of these modalities in different gastrointestinal diseases based on evidence from the literature. We will also highlight the challenges and future implications that might enhance the industrial and clinical practices of this field.

### MATERIALS AND METHODS

The design of this study is a literature review aiming to provide comprehensive evidence and a discussion about the applications and challenges of cellular therapy in gastrointestinal diseases. We aimed to be as comprehensive as possible. Therefore, we conducted an electronic search over different databases, including Google Scholar, Embase, PubMed, Scopus, and Web of Science. We used the search terms (gastrointestinal OR intestinal OR GIT OR inflammatory bowel disease OR IBD OR “Crohn’s disease” OR “ulcerative colitis” OR “Coeliac disease” OR “intestinal graft versus host disease OR GvHD) AND (“Stem cell” OR “cellular therapy” OR “hematopoietic stem cells” OR HSCs OR “mesenchymal stem cells” OR MSCs) on January 2, 2023, to retrieve all relevant articles that are published in English and investigate the applications of cellular therapy in gastrointestinal diseases. We also searched the Cochrane databases and Clinicaltrial.gov to retrieve any ongoing trials in the same context. No restrictions regarding the country or year of publication were applied to be as comprehensive as possible.

### Safety of Cellular Therapy

Evidence from various reports investigating cellular therapy for different gastrointestinal diseases indicates its efficacy. However, some reports have raised concerns about the potential risk of developing serious unfavorable events that might overcome the anticipated benefits. Different adverse events can potentially develop secondary to cellular therapy, including the potential transmission of adventitious agents, immunogenicity, and tumorgenicity.^7^ Different factors, like the extrinsic risk associated with the manufacturing process, intrinsic cell attributes, and other clinical considerations, should also be considered when assessing the overall risk of these modalities.^7^ Accordingly, the risk of developing adverse events might be remarkably variable based on the cell therapy product. For instance, treatment with pluripotent stem cells such as iPSC might be associated with an increased risk of teratoma formation because of the increased number of undifferentiated cells within the final product.^8^ In the following section, we will further discuss the adverse events associated with hematopoietic stem cell therapy (HSCT) and MSCs.

For instance, allogenic HSCT might increase the risk of serious adverse events, like graft rejection, although it has been demonstrated that this modality can significantly treat inflammatory bowel disease (IBD). Autologous HSCT, on the other hand, is usually associated with increased potential of long-term remission due to absent triggers following immune system rebuild since it does not principally target the genetic defect.^6^ Therefore, the safety and success of stem transplantation are usually determined by different factors, like cellular transformation during in vitro expansion, the potential of allogenic tissue formation, the safety of cell culture, and the immunogenicity of stem cells. In this context, a review of data from clinical trials published between 1997 and 2009 on HSCT for immune-mediated disorders by Snowden et al^9^ showed that the 1- and 5-year survival rates of allogenic HSCT were 87% and 65%, compared to 85% and 78% for autologous HSCT, respectively. Various adverse events were reported, and the most common cause of death was infection. These findings indicate that the safety of HSCT in treating immune-mediated conditions, including gastrointestinal ones, is a major concern that should be further investigated in future trials.

Mesenchymal stem cell safety is still inconclusive in the current literature, as well. Based on the multidirectional and proliferative differentiation abilities of MSCs, evidence shows that the risk of tumorigenesis might increase with these therapeutic modalities. A previous animal investigation also showed that tumor appearance and prominence occurred earlier than anticipated when tumor cells were simultaneously infused with adipose-derived stromal cells (ASCs).^10^ On the other hand, some reports indicated that MSCs might inhibit tumorigenesis. For instance, Chen et al^11^ concluded that MSCs decrease the risk of colitis-related tumor formation secondary to different mechanisms, including reduced expression of pro-inflammatory factors, reduced inflammation at the bulk level, downregulation of STAT3 phosphorylation expression, and reduced tumor load and number. Moreover, Nasuno et al^12^ showed that MSCs could significantly suppress tumor formation because of their abilities to control cell division and induce apoptosis.

It has been furtherly shown that MSCs might be associated with an increased risk of developing other systemic adverse events other than carcinogenicity. In this context, a systematic review by Lalu et al^13^ investigated the frequency of adverse events reported following the systemic use (venous and arterial injections) of MSCs. The authors demonstrated that the modality is safe with no serious adverse events, except for the potential development of transient fever.

Reducing the risk of adverse events associated with cellular therapy should be based on a collaborative approach between healthcare authorities, industry, and academia to maximize the benefits of these modalities. Accordingly, it is essential to develop protocols to alleviate the security of these products from manufacturing to clinical use to decrease the potential risks and avoid any obstacles that might intervene against rapid clinical applicability.^14,15^

## Applications and Efficacy of Cellular Therapy in Gastrointestinal Diseases

### Applications of Mesenchymal Stromal Cells

#### Coeliac Disease:

Some studies investigated the efficacy of cellular therapy for managing the coeliac disease. For instance, evidence shows that MSCs might potentially reduce the pathogenic mechanisms involved in developing coeliac disease, including reduction of antigen presentation by dendritic cells, inhibition of T-cell proliferation, and inducing cytoprotective effect on the gut epithelial barrier by altering the balance between anti- and pro-apoptotic factors.^16,17^ However, there are no currently available human trials or published clinical data about using MSCs to treat coeliac disease.

#### Intestinal Graft versus Host Disease:

Hematopoietic stem cell therapy can be associated with various complications, including GvHD, which might also result secondary to solid organ transplantation, like liver transplantation. Steroid therapy is the main line of treatment. However, it has been estimated that around 40% of patients using steroids do not have favorable outcomes.^5^ Accordingly, various clinical trials have investigated the efficacy of MSCs for these patients. For instance, in a case report, third-party haploidentical MSC was effectively used in a pediatric case with severe steroid-resistant GvHD.^18^ However, findings from subsequent clinical trial data show mixed results. For instance, a double-blinded phase III clinical trial demonstrated that the efficacy of prochymal allogenic MSCs did not significantly differ from the placebo in managing GvHD.^19^ On the other hand, data from phase II trials showed that using MSCs for treating severe steroid-refractory GvHD following human leukocyte antigen (HLA)-haploidentical stem cell transplantation.^20^ These also indicated the efficacy of MSCs as a prophylactic modality against developing GvHD.^6,21^ It should be noted that these favorable outcomes do not depend on the source of MSC, including HLA-mismatched and identical donors.^20^ A meta-analysis by Hawkey and Hommes^22^ aimed to make a better conclusion about the efficacy of MSCs for managing GvHD. They found that the efficacy of MSCs in GvHD prophylaxis is uncertain, and further trials are needed. However, evidence shows that using prochymal MSCs has been approved in New Zealand, Canada, and the USA.

#### Inflammatory Bowel Disease:

Mesenchymal stromal cells in Crohn’s disease was previously proven effective in managing peri-anal fistulas.^23^ Accordingly, it has gained remarkable attention for managing IBD, and various RCTs were conducted in this context (Table 2).^24-46^ These multi-phasic large sample-size clinical trials have investigated the efficacy and safety of MSC in perianal fistulas and Crohn’s disease.^47^ For instance, a multicenter phase III RCT (ADMIRE CD) investigated the efficacy of allogeneic expanded adipose-derived MSC (Cx601) as a single local injection of 120 million cells for managing perianal Crohn’s disease compared to a placebo group. The trial included 212 patients from 49 hospitals. The findings indicated that combined remission, including absent collections of the treated perianal fistulas and closure of all treated external openings draining at baseline at 24 weeks, was successfully achieved in patients receiving MSC.^23^ In 2022, the authors furtherly demonstrated the well-tolerability of MSCs and that clinical remission can be maintained for 104 weeks post-treatment in patients with perianal fistulizing Crohn’s disease.^48^ Although the efficacy and safety of MSC have been indicated to be favorable among different clinical trials, some limitations should be considered before considering MSC for managing IBD. For instance, there is significant heterogeneity among the published clinical trials, which might cause bias in interpreting the reported findings. Accordingly, some factors should be considered when conducting future clinical trials to enhance the quality of evidence. These might include defining the optimal route of administration and dose, rigorous sorting, and cell selection (autologous versus allogeneic) to reduce heterogeneity and enhance evidence for the long-term effectiveness of MSC for prolonged remission.^5,6^

It is also important to consider the administration of immunosuppressive treatment modalities in combination with MSC for managing IBD. It would be vital to investigate whether such combined administration might impact the efficacy of MSC in these patients. In this context, Dujivestein et al^49^ reported that using MSC in combination with immunosuppressive therapy, including anti-TNF-α, 6-MP, methotrexate, and azathioprine, in IBD patients, did not impact survival, phenotype, immunosuppressive, and differentiation capacity. It was also reported that the immunomodulatory effects of MSC were even better in combination with anti-TNF-α and 6-MP antibodies, suggesting that such a combination might have better outcomes. Various clinical trials furtherly investigating the various aspects of MSC therapy for IBD have been registered (Table 3).

### Applications of Hematopoietic Stem Cell Therapy

#### Coeliac Disease:

It has been demonstrated further that HSCT might be effective for managing coeliac disease and are particularly suitable for patients with enteropathy-associated T-cell lymphoma (EATL) and others who do not respond to gluten-free diets.^50^ In 2007, Al-toma et al^51^ indicated this as the authors reported using HSCT following conditioning with melphalan and fludarabine for managing 7 cases suffering from refractory coeliac disease II (with aberrant intraepithelial lymphocytes). The authors showed that patients receiving HSCT modalities showed a significant normalization in biochemical and hematological biomarkers and decreased aberrant T cells within the duodenal biopsies of these patients. Besides patients with refractory coeliac disease, it has been further shown that HSCT can be effectively used among others with associated hematological life-threatening conditions.^52-54^ These studies indicated that HSCT was significantly associated with reduced antigen-specific CD4+ T-cell memory in vivo responses and in vitro unresponsiveness to gluten, although gluten was introduced into the diet. 

However, these studies are limited by the small sample size and short follow-up periods, indicating the need for further validation of the current promising findings. It should also be noted that not all the currently available data about using HSCT for coeliac disease patients are promising. For instance, Al-toma et al^55^ demonstrated that their findings do not support using of HSCT among patients with EATL. This is because 3 of their population with EATL died within the treatment period with HSCT.

#### Inflammatory Bowel Disease:

The initial indication for conducting HSCT in IBD was the presence of combined hematological complications (like non-Hodgkin’s lymphoma and leukemia). However, further indications were reported in the clinical settings, particularly after the indicated favorable outcomes in improved intestinal lesions during transplantation. Hematopoietic stem cells can be obtained from bone marrow, peripheral blood, and core blood, which directly migrate into the injured tissues and differentiate into immunomodulatory and epithelial cells that can effectively restore the normal functions of the injured mucosal cells.^56^ Applying HSCT is usually multimodal since it requires adequate preparation to obtain enhanced outcomes. For instance, pre-transplant screening includes bone marrow aspiration, small bowel, pelvic, or rectal MRI, colonoscopy, serology, blood tests, and taking adequate history and physical examination.^57^ First, stem cell mobilization from the human leukocyte antigen (HLA)—the matched donor is required following bone marrow stimulation to induce the production of stem cells after removing lymphocytes by infusion cyclophosphamide. Following leukocyte clearance, it is recommended that CD34^+^ should be collected from the bone marrow or peripheral blood at a final count of 3-8 ×10^6^. Eventually, transplantation and reconstruction of the immune system are conducted.^6,58,59^

Applying HSCT for treating IBD was first reported in the 1990s, and evidence shows that autologous HSCT was preferred over allogenic HSCT to reduce the risk of developing GvHD.^60^ Little research can be found in the literature regarding the efficacy and safety of HSCT in patients with ulcerative colitis (Table 2). However, some studies with limited sample sizes investigate the outcomes of patients receiving HSCT for ulcerative colitis combined with hematological malignancies.^61,62^ The findings of these investigations indicate the efficacy of HSCT on disease remission. Moreover, it has been concluded that HSCT should not be contraindicated in patients with ulcerative colitis since it has been found that the latter did not increase the risk of GvHD in this population. However, the morbidity and mortality associated with HSCT should make clinicians and healthcare practitioners reconsider applying HSCT for ulcerative colitis as the only management approach.

On the other hand, data regarding the application of HSCT for managing Crohn’s disease can be found in the literature since some large-center RCTs have been published in this regard. For instance, in 2015, autologous HSCT was used for treating refractory Crohn’s disease in a multicenter phase III RCT, the ASTIC trial.^63^ The authors demonstrated that the treatment group had significantly enhanced outcomes than the control one. For instance, a reduced need for any therapeutic modality was noticed among 61% and 23% of the HSCT and control-treated groups (*P* < .01), respectively, at 3 months of follow-up. Moreover, it has been found that the rate of disease remission at radiology and endoscopy was 35% in the HSCT and 6% in the control groups (*P* = .053).

Results from the European Society for Blood and Marrow Transplantation retrospective study demonstrated that 43% of 82 Crohn’s patients treated with HSCT achieved clinical remission at 1 year.^64^ However, it was also shown that reintroducing conventional therapy was required in 73% of these patients after a median of 10 months. It should be noted that there is limited data about the mechanism of the reported clinical efficacy of HSCT in IBD.^65,66^ In this context, a multi-center RCT in the UK (ISRCTN17160440) is currently recruiting IBD patients to investigate whether the intensity of HSCT in these patients is somehow associated with the incidence of adverse events.

### Applications of T-Cell Therapy (T-regs)

#### Intestinal Graft versus Host Disease:

Managing GvHD with T-cell therapy shows promising results. For instance, pre-clinical studies demonstrated the favorable outcomes that might be obtained from thymic-derived T-regulatory cells (T-regs) in treating chronic GvHD and preventing the development of acute disease.^67-70^ On the other hand, data regarding peripheral-derived T-reg were not encouraging in this regard, which might be attributed to the phenotypic instability characteristics of these modalities, leading to loss of FOXP3 expression following transplantation.^71^ In this context, we found an ongoing RCT (NCT03577431) that aims to investigate the efficacy of mixed leukocyte reaction-generated allospecific T-regs combined with donor-specific cells in preventing GvHD (facilitating early immunosuppression withdrawal) among patients with liver transplantation.

#### Inflammatory Bowel Disease:

The efficacy of T-regs for managing inflammatory conditions, including IBD, was investigated in murine models of experimental studies. These studies indicate the adoptive transfer of these modalities, leading to significant prevention of inflammation.^72^ However, this evidence lacks strengthening clinically by data from RCTs. In 2012, Desreumaux et al^73^ conducted the CATS1 trial (a phase I/IIa open-labeled clinical study) and recruited 20 patients with Crohn’s disease to investigate the efficacy of intravenous administration of T-reg therapy obtained from ovalbumin-specific T-reg cells isolated from patient’s peripheral blood mononuclear cells. The authors demonstrated that the efficacy was dose related, the injections were well-tolerated, and 40% of the patients had >100 points of Crohn’s disease activity index (CDAI) reduction at the fifth and eighth weeks. However, it has been demonstrated that single-dose clinical effects were of short-term and that only clinical remission was achieved in 10% of the population (CDAI < 150).

Based on the favorable findings from the CATS1 study, another phase IIb trial was conducted, and the results were anticipated to be published in 2018 (NCT02327221). However, the trial was stopped due to manufacturing issues regarding T-reg therapy. Moreover, some adverse events were reported, including the development of an anaphylactic reaction in 1 included patient. Therefore, no adequate data could be collected from this trial, and no clear conclusions could be made regarding T-regs’ efficacy in managing IBD. Another RCT by Goldberg and colleagues also investigated the efficacy of T-reg cells isolated from lamina propria or peripheral blood or of patients with Crohn’s disease compared to the control group. Based on in vivo and in vitro findings, the authors demonstrated that these modalities constitute an optimal therapeutic issue for Crohn’s disease.^74^

### Applications of Embryonic Stem Cells

**Figure 1.**
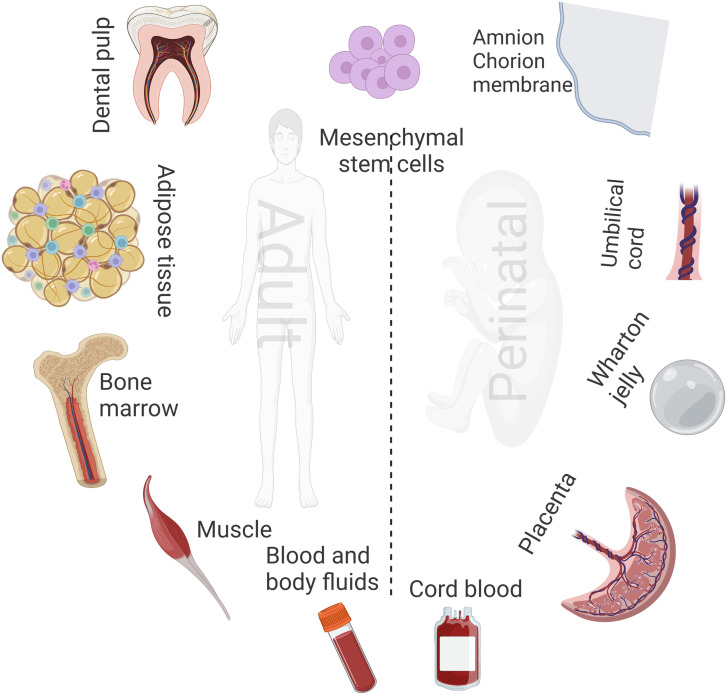
Different sources of embryonic and adult mesenchymal stem cells.

Embryonic stem cells can be obtained from different perinatal sources (Figure 1). Embryonic stem cells have been widely used and have gained attention in various clinical applications. They are embryo-derived pluripotent and self-renewing cells that can effectively differentiate into intestinal immune and epithelial cells.^75^ Furthermore, these cells can differentiate more extraordinarily and have a faster growth rate than adult stem cells. They also have inflammation-relieving and immunomodulatory characteristics and can even differentiate into other stem cells that might be used for treating IBD.^76^ However, data from the current literature is too minimal to make conclusions about the efficacy of using these modalities in managing IBD. An animal investigation by Srivastava et al^77^ used pyrrolidone-induced colitis in IL-10^−us^ KO mice models to investigate the efficacy of mouse ESC transplantation on immune imbalance and the severity of colitis. The authors reported that the underlying induced inflammation was significantly relieved by reconstructing the immune system and repairing the damaged epithelium as the transplanted ESCs effectively colonized the colon, small intestine, and liver. The clinical application of these modalities in IBD remains a major challenge since it is difficult to control the differentiation characteristics of these modalities and fit them into treating and preventing the characteristic disease pathways of IBD.

### Current Challenges and Future Implications

Cellular therapy has proven its efficacy in managing various gastrointestinal diseases. It has a promising future in this field, with HSCT, MSCs, and T-reg cells being remarkable candidates due to their proven immunomodulatory effects. It should be noted that HSCT has recently been introduced, especially for managing hematological malignancies, and investigating its capabilities in different fields has increased since 2013. However, only 0.1% and 1% of allogeneic and autologous HSCT are performed in relation to autoimmune diseases in Europe.^78^ This shows that the techniques for applying HSCT in autoimmune diseases are still largely based on the ones established for hematological malignancies.

Overcoming safety-related concerns is also a challenge for efficaciously applying these modalities for gastrointestinal diseases. The main adverse events related to HSCT application are GvHD and infections, with an estimated incidence of 11%-18% and a subsequent mortality risk of 70%-90% in severe events.^79^ Therefore, applying HSCT in gastrointestinal diseases might be limited to severe life-threatening refractory cases of Crohn’s disease. The efficacy of HSCT depends on many factors, including choosing the conditioning regimen, the type of disease, and the source of cells. Accordingly, it is vital to make the conditioning regimens optimal and choose the most compatible patient to benefit from the procedure.

#### Immunogenicity of MSC:

MSCs might be favored over HSCT by the relative absence of immunogenicity, which decreases the need for total body irradiation and chemotherapy. This has been shown in most clinical trials where administered MSC was allogeneic without needing immunosuppression or HLA matching. However, it is controversial whether MSCs have immunogenicity or not. Mesenchymal stem cells do not induce a T-cell response in mixed lymphocyte reactions because they do not express key co-stimulatory and MHC class II molecules that activate T-cells.^80^ However, it has been evidenced that clearing MSCs from the body rapidly occurs following infusion. Moreover, data from clinical trials indicate that alloantibodies were detectable in some patients infused with allogenic MSC.^81^ Another theory suggested that lack of hemocompatibility might induce an innate immune reaction following MSC infusion independent of HLA matching disparity. In a study investigating the efficacy of pancreatic islet cell infusion, the authors showed that around 80% of the cells were lost due to the instant blood-mediated inflammatory reaction (IBMIR).^82^

Instant blood-mediated inflammatory reaction-related cellular destruction is mediated by the coagulation/complement activation pathway. Therefore, the absence of immunogenicity secondary to MSCs should be reconsidered, although immune reactions with these modalities are usually slower than the ones observed with HSCT. However, it is still not clear with such immunogenicity impacts the efficacy of MSC therapeutic efficacy. In this context, immunosuppression-related MSC apoptosis in vivo was evidenced by Galleu et al.^83^ Le Blanc et al^84^ conducted a phase II clinical trial and showed the therapeutic efficacy between allogeneic and autologous MSCs was similar. However, there is a lack of data regarding antibody response after the infusion of these modalities. Although it has not been evidenced yet alloantibodies formation following MSCs might suggest that repeated infusion might reduce their therapeutic efficacy. This has been indicated by Dang et al^85^ which showed that the immunomodulatory effects of MSC were impacted by MSC apoptosis. On the other hand, de Witte et al^86^ showed that MSCs usually accumulate in the lungs after infusion and then undergo apoptosis and phagocytosis by monocytes. It has been suggested that such phagocytosis stimulates the host monocytes to initiate an immunomodulatory phenotype, enhancing the beneficial effects of MSCs.^86^

#### T-regs Concerns:

T-reg cells are also promising, with efficacious findings regarding their use in managing immune-mediated disease. However, the outcomes of patients with immune-mediated diseases might not be favorable if the phenotype of T-reg cells changes following administration (for instance, to Th17 cells) or if the T-cell population was contaminated by effector T-cells. Obtaining pure T-reg cells by stringent use of standardized culture conditions might, therefore, decrease the risk of anticipated adverse events. The treatment strategy should also be based on choosing specific T-reg subtypes according to their functional characteristics. Canavan et al^87^ isolated T-regs (CD4 + CD25 + CD127loCD45RA- and CD4 + CD25 + CD127loCD45RA+) from a patient with Crohn’s disease and tested it in a human intestinal xeno-transplant model after being expanded in vitro. The authors demonstrated that CD45RA+ T-reg in vitro conversion into Th17 cells was not observed and, instead, it induced the expression of different molecules, like α4β7 integrin, that facilitated gut homing. The efficacy of T-reg can also be determined by efficient homing when targeting damaged organs. For instance, Scotta et al^88^ compared the efficacy of cord blood and adult T-regs in treating GvHD in vivo and in vitro using a model with human skin transplantation. They showed that in vitro immunomodulatory activities were comparable between both sources. However, preventing GvHD was achieved in vivo only by using adult T-regs with skin-compatible homing receptors.

#### Limitations to Current Clinical Trials:

Limitations found in clinical trials should also be considered to enhance the quality of available evidence and boost the efficiency of clinical practice. For instance, there is significant heterogeneity among the currently available trials regarding study design, injection route, organ source, and injected dosage.^89^ Therefore, the optimal strategy for MSCs remains unknown. Moreover, it is difficult to pool current data due to the heterogeneity among the available trials. Hematopoietic stem cells and MSCs can be obtained from different sources (e.g., adipose tissue and bone marrow) with no evidence regarding the superiority of any of these over the other. Accordingly, future investigations should also investigate the best sources that might be optimal for application in clinical practice.

**Figure 2.**
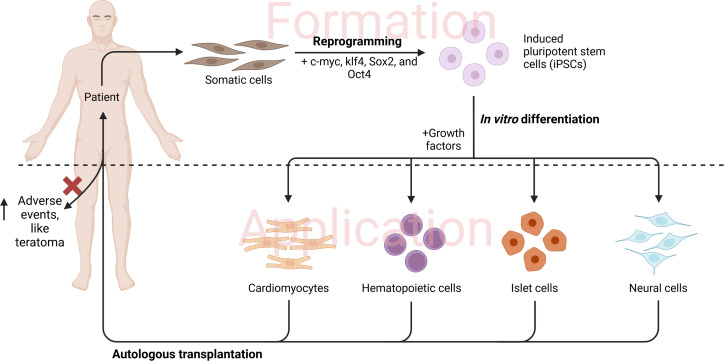
Formation and application of induced pluripotent stem cells.

Another major limitation reported among clinical trials of T-reg therapy is the isolation of adequate functional cells. However, overcoming such a limitation can be surpassed by using induced pluripotent stem cells (iPSCs). Induced pluripotent stem cells can be generated by using specific transcription factors (c-myc, klf4, Sox2, and Oct4) to induce mature specialized cells into embryonic-like cells,^90^ which can then be differentiated into the targeted cells by manipulating growth factors (Figure 2). Functional T-regs were successfully generated by Haque et al^91^ from iPSCs. However, using such approaches in clinical practices is still limited due to ethical considerations and technique hurdles, together with the increased risk of teratoma formation with iPSCs. Induced pluripotent stem cells can alternatively be used for tissue regeneration in areas with inflammation-related gastrointestinal tissue damage, although extensive future work is needed before making this clinically valid.

## Conclusion

The future for applying cellular therapy in gastrointestinal diseases is promising. Remarkable advances have been reported in research studies. These investigations indicated the efficacy of HSCT, MSCs, and T-regs in the management of gastrointestinal diseases. However, various considerations to applying these modalities in gastrointestinal diseases exist, and future studies should aim at enhancing the current challenges and limitations to using cellular therapy. For instance, the application of HSCT needs technically advanced settings and extensive pre-application screening to intervene and manage any post-application adverse events and complications.

Despite the promising findings, cellular therapy remains a novel technique that needs to be furtherly studied to comprehend various factors associated with its application in gastrointestinal diseases. For instance, limitations regarding immunogenicity and adverse events of stem cells, and others regarding the purification and isolation process should be addressed.^92^ Moreover, future studies should aim at understanding the long-term efficacy and safety, the mechanism of cellular therapy to boost the therapeutic potential of these modalities in the different immune-mediated and inflammatory intestinal diseases. Optimizing the factors that might affect the efficacy of cellular therapy should also be investigated by future studies to enhance the process of application and patient selection. Anticipated findings from the ongoing phase III clinical trials might overcome these challenges and provide insight standardized application of stem cells. Using cellular therapy by other modalities, rather than HSCT and MSCs, should also be encouraged and investigated by preclinical models.

## Figures and Tables

**Table 1.** A Comparison between the Different Therapeutic Approaches for Inflammatory Bowel Diseases

Characteristics	Mesenchymal Stem Cells	Hematopoietic Stem Cells	mAb therapy	Immunosuppressive Drugs	Immune Cell Therapy	Conventional Pharmacological Treatment
Mechanism of action	Differentiation into various cell types, secrete anti-inflammatory cytokines, and modulate immune responses	Differentiation into various cell types, secrete cytokines, and modulate immune responses	Bind to specific antigens on the surface of cells to block their activity	Suppress the activity of the immune system by blocking certain pathways	Introduce new immune cells to replace those that are damaged or destroyed by disease	Target specific molecules involved in disease processes to reduce inflammation and other symptoms
Advantages	Can be used in a variety of diseases and conditions; can be used for regenerative purposes; can be used to reduce inflammation; can be used to restore tissue function	Can be used in a variety of diseases and conditions; can be used for regenerative purposes; can be used to reduce inflammation; can be used to restore tissue function	Highly specific targeting of antigens; less risk of side effects than other treatments; long-term efficacy with minimal dosing requirements	Can reduce inflammation quickly and effectively; fewer side effects than other treatments; long-term efficacy with minimal dosing requirements	Can replace damaged or destroyed cells with healthy ones; may provide long-term protection from disease recurrence or progression	Quickly reduces inflammation and other symptoms associated with IBD; fewer side effects than other treatments; long-term efficacy with minimal dosing requirements
Disadvantages	Risk of tumor formation if not properly monitored or regulated; potential for immunological rejection if not properly matched with patient’s tissue type	Risk of tumor formation if not properly monitored or regulated; potential for immunological rejection if not properly matched with patient’s tissue type; limited availability due to donor shortage	Potential for adverse reactions due to non-specific binding of antibodies to healthy cells/tissues; limited availability due to cost and production time	Potential for adverse reactions due to non-specific suppression of the immune system; potential for drug resistance over time	Limited availability due to donor shortage; potential risk of introducing new pathogens into the body; potential risk of introducing new autoimmune diseases into the body	May require multiple doses over a long period of time; may cause undesirable side effects such as nausea, vomiting, diarrhea, etc.; may not provide long-term protection from disease recurrence or progression

**Table 2.** Current Trials Investigating the Efficacy and Safety of Stem Cell Therapy in Patients with Inflammatory Bowel Diseases

References	Year	Included Patients		Trial Design/Phase	Stem Cell			Author Conclusion
		Type	n		Type	Dosage	Route	
Cho et al^24^	2013	CD	10	I	Autologous AD–MSC	2-4 × 10^7^	Local/Intrafistular	Reduced inflammation and complete healing of fistula
Cho et al^25^	2015	CD	43	II	Autologous AD–MSC	3 × 10^7^	Local/Intrafistular	Effective in fistula healing and safe treatment
Ciccocioppo et al^26^	2011	CD	12	ND	Autologous BM–MSC	20 × 10^6^	Local/Intrafistular	Increased T-reg cells and reduced CDAI
De la Portilla et al^27^	2013	CD	24	I/IIa	Allogenic AD–MSC	20 million	Local/Intrafistular	MRI index improvement and complete closure of the fistula
Dhere et al^28^	2016	CD	12	I	Autologous BM–MSC	10 million/kg	Intravenous	Allogenic PBMC was prevented by BM-MSC, which expressed IDO, and clinical improvement was noted.
Duijvestein et al^29^	2010	Refractory CD	10	I	Autologous BM–MSC	1e2310^6^ cells/kg	Intravenous	Two patients had decreased CDAI, and MSC therapy was considered feasible and safe.
Forbes et al^30^	2014	Luminal CD	16	Open labeled (II)	Allogenic BM–MSC	2 × 10^6^ cell/kg	Intravenous	Endoscopy improvement, clinical remission, and reduction in CDAI were noted in 7/12, 8/12, and 12/12 patients
Gregoire et al^31^	2018	Refractory luminal CD	13	Open labeled (I-II)	Allogenic BM-MSC	Two injections of 1.5-2.0 × 10^6^ cell/kg	Intravenous	No adverse events were reported. CDAI significantly decreased and 4 patients finally achieved clinical remission.
Guadalajara et al^32^	2012	CD	49	II	Autologous AD-MSC + fibrin glue		Local/Intrafistular	No relapse occurred in 7 patients and treatment was safe on a long-term follow-up basis
Herreros et al^33^	2012	CD	200	III	Autologous AD-MSC	20 million	Local/Intrafistular	Fistula healing was achieved in 40% of patients and the treatment was safe
Hommes et al^34^	2011		3	ND	Autologous HSC	3.5-5.9 × 10^6^/kg	Intravenous	Clinical remission was achieved
Hu et al^35^	2016	UC	40	I/II	Allogenic UC–MSC	3.8 ± 1.6 × 10^7^	Intravenous	No significant changes were noted in IFN-γ, TNF-α, and IL-6, and Mayo score significantly improved.
Lee et al^36^	2013	CD	43	II	Autologous AD–MSC	3 × 10^7^	Local/Intrafistular	Complete healing of fistula
Liang et al^37^	2012	CD/UC	7	I	Allogenic BM–MSC/UC–MSC	1 × 10^6^ /kg	Intravenous	Effective and safe treatment
Lightner et al^38^	2022	Refractory UC	6	IB/IIA randomized control clinical trial	Allogenic BM-MSC	150 million cells	Local/Intrafistular	Improved endoscopic and clinical outcomes
Mayer et al^39^	2013	CD	12	I	Allogenic Placenta-derived MSC	2-8 × 10^8^	Intravenous	Treatment was safe and remission was achieved in the low-dose group.
Molendijk et al^40^	2015	CD	21	randomized, double-blind, dose-escalating clinical trial	Allogenic BM-MSC	1 × 10^7^ 3 × 10^7^ 9 × 10^7^	Local/Intrafistular	Fistula healing in 85.6% of patients who received 3 × 107 cells with no adverse events
García-Olmo et al^41^	2005	CD	5	I	Autologous AD-MSC	3–30 × 10	Local/Intrafistular	Discharge decreased and fistula healed significantly
García-Olmo et al^42^	2009	CD	50	II	Autologous AD–MSC		Local/Intrafistular	Fibrin glue was less effective than stem cell therapy in fistula healing
Panés et al^23^	2018	CD	212	Double blind (III)	Allogenic AD-MSC	Single dose of 120 million cells	Local/Intrafistular	Clinical remission was achieved in 59.2%
Ruiz et al^43^	2017	Refractory CD	14		Autologous HSCT	4.3 – 36.7 × 10^7^	Intravenous	Four patients developed complications, and CDAI was significantly reduced
Vieujean et al^44^	2022	CD	10	I-II	Allogenic BM–MSC		Local/Intrafistular	5/10 achieved complete resolution in the 12th week and 7 in the 48th week.
Wainstein et al^45^	2018	CD	9	I	AD–MSC + PRP	100-120 million	Local/Intrafistular	Improved activity index and complete fistula closure
Zhang et al^46^	2018	CD	82	RCT	Allogenic UC–MSC	1 × 10^6^ /kg	Intravenous	No complete remission was achieved with significant improvement in fistula closure, endoscopic index, and CDAI reduction

AD, adipose tissue; BM, bone marrow; CD, Crohn’s disease; CDAI, Crohn’s disease activity index; HSCT, hematopoietic stem cell therapy; IDO, indoleamine 2,3-dioxygenase; MSC, mesenchymal stem cell; PBMC, peripheral mononuclear cells; PRP, platelet-rich plasma; RCT, randomized controlled trial; UC, ulcerative colitis.

**Table 3.** Registered Clinical Trials Investigating the Efficacy and Safety of Mesenchymal Stem Cell Therapy for Inflammatory Bowel Disease Patients

Country	Phase	MSC Source	Patients	Recruitment	Registration Number
United States	I	Allogenic BM–MSC	Pediatric inflammatory bowel disease	Completed not published	NCT02150551
Jordan	I	Allogenic Warton’s jelly MSC	UC	Unknown	NCT03299413
United States	I	Autologous BM–MSC	UC	Completed not published	NCT01659762
Belgium	I	MSC	CD	Recruiting	NCT03901235
China	II	MSC	CD	Not yet recruiting	NCT03056664
United States	I	Autologous MSC	CD	Recruiting	NCT03449069
China	I	Allogenic AD–MSC	UC	Recruiting	NCT03609905
China	I	Allogenic UC–MSC	UC	Unknown	NCT02442037
Iran	I	Autologous BM–MSC	CD	Unknown	NCT01874015
United States	II	Allogenic BM–MSC	CD	Completed not published	NCT00294112
Italy	II	Autologous AD–MSC	CD	Unknown	NCT02403232
United States	III	Allogenic MSC (PROCHYMAL)	CD	Completed not published	NCT00482092
Spain	I-II	Allogenic AD–MSC	UC	Unknown	NCT01914887
Spain	I	Autologous AD–MSC	CD	Completed not published	NCT01157650
United States	III	Allogenic MSC (PROCHYMAL)	CD	Completed not published	NCT00543374
Korea	I	Allogenic AD–MSC	CD	Unknown	NCT03183661
South Korea	I	Allogenic AD–MSC	CD	Recruiting	NCT02580617
United States	II	Autologous AD–MSC	CD	Unknown	NCT02403232

AD, adipose tissue; BM, bone marrow; CD, Crohn’s disease; HSCT, hematopoietic stem cell therapy; MSC, mesenchymal stem cell; UC, ulcerative colitis.
